# Estradiol Ameliorates Acute Kidney Ischemia-Reperfusion Injury by Inhibiting the TGF-βRI-SMAD Pathway

**DOI:** 10.3389/fimmu.2022.822604

**Published:** 2022-02-24

**Authors:** Lian Ren, Fang Li, Ziyang Di, Yan Xiong, Shichen Zhang, Qing Ma, Xiaoen Bian, Zhiquan Lang, Qifa Ye, Yanfeng Wang

**Affiliations:** ^1^ Zhongnan Hospital of Wuhan University, Institute of Hepatobiliary Diseases of Wuhan University, Transplant Center of Wuhan University, Hubei Key Laboratory of Medical Technology on Transplantation, Wuhan, China; ^2^ Department of General Surgery, Taihe Hospital, Hubei University of Medicine, Shiyan, China; ^3^ Eye Center, Renmin Hospital of Wuhan University, Wuhan, China; ^4^ Department of Gastrointestinal Surgery & Department of Gastric and Colorectal Surgical Oncology, Zhongnan Hospital of Wuhan University, Wuhan, China

**Keywords:** estradiol, ERα, TGF-βR1, SMAD, ischemia-reperfusion injury

## Abstract

Renal ischemia–reperfusion injury (IRI) is less extensive in females than males in both animals and humans; however, this protection diminishes after menopause, suggesting that estrogen plays a pivotal role in IRI, but the underlying mechanism remains largely unknown. Our study found that 45 min of warm ischemia was sufficient to induce significant pathological changes without causing death in model animals. Compared with male rats, female rats exhibited less extensive apoptosis, kidney injury, and fibrosis; these effects were worsened in ovariectomized (OVX) rats and ameliorated upon estradiol (E_2_) supplementation. Furthermore, the levels of TGF-βRI, but not TGF-βRII or TGF-β1, were significantly increased in OVX rats, accompanied by phosphorylated SMAD2/3 activation. Interestingly, the alteration trend of the nuclear ERα level was opposite that of TGF-βRI. Furthermore, dual luciferase reporter and chromatin immunoprecipitation assays showed that ERα could bind to the promoter region of TGF-βRI and negatively regulate its mRNA expression. Moreover, an *in vitro* study using NRK-52E cells showed that ERα knockdown blocked E_2_-mediated protection, while TGF-βRI knockdown protected cells against hypoxic insult. The findings of this study suggest that renal IRI is closely related to the TGF-βRI-SMAD pathway in females and that E_2_ exert its protective effect *via* the ERα-mediated transcriptional inhibition of TGF-βRI expression.

## Introduction

Renal ischemia–reperfusion injury (IRI), which occurs in 30%–50% of transplant patients, is a prevalent risk factor that contributes to acute kidney injury (AKI) and delayed graft function after kidney transplantation (1). As there are no effective treatments for AKI, renal IRI is a major cause of morbidity and mortality (2) and accounts for 1% of hospital admissions, and its prevalence is approximately 7% in hospitalized patients (3).

Female animals are less likely to suffer from AKI than males (4), and estrogen protects renal tubular function after experimental ischemic injury (5). Male animals, including humans, are more susceptible to IRI-induced kidney injury with worsened renal function than females (6). Previous studies reported that the mortality rates of male rats and mice were significantly increased in a renal IRI model, and renal function recovery and tissue injury were shown to be sex dependent due to sex hormone production (7). A retrospective cohort study of AKI complicating acute myocardial infarction-related cardiogenic shock mentioned that women with AKI were older, which suggested the important role of estrogen in AKI (8). A systematic review of dialysis practices among men versus women with AKI suggested that men were more likely to develop AKI and require renal replacement therapy (RRT) than hospitalized women (9). Moreover, a clinical study suggested that menopause contributes to more renal dysfunction in women due to estrogen withdrawal (10). Furthermore, the application of estrogen-based postmenopausal hormone replacement therapy (HRT) has been suggested to ameliorate renal function in postmenopausal women and delay chronic kidney disease (CKD) progression (11). However, until now, the mechanisms responsible for the sex hormone-mediated differences in humans and animals with AKI have not been fully explored.

Transforming growth factor-β1 (TGF-β1) is the primary factor that drives fibrosis in patients with CKD (12). After it is released, active TGF-β1 binds to its type II receptor (TGF-βRII) and then transphosphorylates and activates the type I receptor (TGF-βRI) and downstream SMAD2 and SMAD3 to regulate genes associated with renal fibrosis (13). Several studies have suggested a close association between the TGF-β1 receptor and AKI. Conditional knockout of TGF-βRII in mice attenuated renal damage by reducing tubular apoptosis under the condition of mercuric chloride-induced injury (14). Another study showed that TGF-βRI overexpression induced renal cell apoptosis, oxidative stress, and interstitial inflammatory cell infiltration (15). Decreasing the expression of TGF-βRI by microRNAs, such as let-7a and miR-140-5p, could effectively ameliorate kidney injury or renal fibrosis by regulating TGF-βR1/SMAD signaling (16, 17). Therefore, as the direct effector of SMAD2/3, TGF-βRI is thought to be the key component of the SMAD signaling pathway (16); however, the role of TGF-βRI in renal IRI is still poorly studied.

Previous studies have demonstrated that estradiol (E_2_) has a renoprotective effect on AKI by regulating the sympathetic nervous system (18) and accelerating renal tubule regeneration (19). Such effects are mainly mediated by estrogen receptors (ERα and ERβ) or G protein-coupled ER (GPER) through genomic or nongenomic mechanisms (20). Analysis of kidney transplantations into recipients of the opposite sex followed by ischemia showed that female recipients had better graft function than male recipients (21), revealing that the sex of the host determined recovery. However, renal IRI was exacerbated in ERα-knockout female mice, while estrogen supplementation before ischemia protected female mice (21). Additionally, ERα-knockout female mice exhibited features of reduced renal growth, including glomerular enlargement at 2 weeks after the induction of streptozotocin-induced diabetes mellitus and compensatory kidney growth at 48 h after uninephrectomy (22). This finding indicated that ERα mediates the protective effect of sex hormones. ERα acts as a transcriptional regulator that activates or inhibits gene expression by directly binding to estrogen response elements (EREs) or by interacting with other transcription factors (23, 24). The administration of estrogen suppresses TGF-β signaling pathways by promoting the degradation of Smad2/3 (25). Additionally, ERα has been implicated in the E_2_-induced rapid activation of ERK1/2 in ERα-transfected cells (26). Although the ERα and TGF-β signaling pathways are major regulators of tissue development, function, and tumorigenesis, they play opposing roles in proliferation and apoptosis (27). TGF-βRI-mRNA-expressing cells were also shown to exhibit ERα immunoreactivity in the anteroventral periventricular nucleus, medial preoptic nucleus, and arcuate nucleus (28). In MC3T3-E1 cells, a clonal preosteoblastic cell line, ERβ is involved in the estrogen-mediated repression of TGF-βRI by binding to EREs located in the TGF-βRI promoter region (29), suggesting a close relationship between ERs and the TGF-βRI/SMAD pathway. As mentioned previously, E_2_ plays a dominant role in AKI, and the mechanisms by which ERs influence the TGF-βRI/SMAD pathway are therefore worthy of further exploration.

Accumulating evidence suggests that estrogen and TGF-βRI/SMAD are involved in AKI regulation. Thus, we investigated whether the ERα signaling pathway interacts directly with the TGF-βRI/SMAD pathway. In the present study, we first compared renal injuries and then investigated the TGF-βRI/SMAD-related pathway in female rats after renal IRI. To further confirm the effect of estrogen, female rats were ovariectomized and then administered E_2_. Furthermore, an *in vitro* cell model was used to explore the mechanism by which ERα regulates the TGF-βRI/SMAD pathway.

## Materials and Methods

### Reagents

17β-E_2_ was purchased from Sigma (St. Louis, MO, USA). TGF-βRI (ab31013, Abcam, Cambridge, MA, USA) and TGF-βRII (346596) polyclonal antibodies were purchased from Zenbio (Chengdu, China). SMAD2 (12584), SMAD3 (9513), pSMAD2 (Ser465/Ser467) (8828), pSMAD3 (Ser423/425) (9520), and ERα (13258) were purchased from Cell Signaling Technology (Danvers, MA, USA). GAPDH (66009-1-Ig) and ATP1A1 (14418-1-AP) were purchased from Proteintech (Wuhan, China). α-SMA (A17910), CoIL (A6428), TGF-β1 (A18692), and Kim-1 (A2831) were purchased from Abclonal (Wuhan, China). Annexin V-FITC/PI Apoptosis Detection Kit (C1062M) and TUNEL-FITC apoptotic kit were purchased from Beyotime (C1088, Haimen, China). TGF-βRI plasmid, TGF-βRIShRNA, and ERα siRNA were purchased from Tsingke (Beijing, China).

### Animals and Groups

Adult male and female Sprague-Dawley (SD) rats weighing 250–350 g were obtained from Wuhan Wan Qian Jia Xing Bio-Technology Co., Ltd. (Hubei, China). Rats were subdivided into following groups: (1) Sham male rat group (male), (2) Sham female rat group (female), (3) male+IRI rat group (male+IRI), (4) female+IRI rat group (female+IRI), (5) ovariectomized (OVX) female+IRI rat group (OVX+IRI), and (5) OVX female+IRI rat-pretreated E_2_ group (OVX+E_2_+IRI). All animals were housed in a temperature-controlled room (22°C ± 2°C) on a 12 h:12 h light-dark cycle with 75% humidity and with standard diet of rat chow and water *ad libitum*. All the procedures used in this study were approved and performed in accordance with the guidelines of the Ethics Committee of the Institute of Wuhan University and conformed to the Guide for the Care and Use of Laboratory.

### Establishment of IRI Animal Model

All surgical procedures were performed under anesthesia with sodium pentobarbitone (60 mg/kg, i.p.). After surgery, ketamine (10 mg/kg, i.p.) was used to control postoperative pain. Two weeks after ovariectomy, OVX+E_2_+IRI rats were given daily subcutaneous injections of 25 μg/kg for 14 days of E_2_ (sesame oil dissolution). The lethal model of rat bilateral nephrectomy was established after both kidneys were surgically removed. After anesthetization, rats were placed on a heat surgical pad (37°C) in a temperature-controlled operative apparatus. Anal temperature was continuously measured throughout and maintained at 36.0°C ± 0.5°C. Using an operation instrument, an abdominal midline incision was made. The right kidney was removed, and the left renal pedicle was exposed and clamped for 45–60 min to induce ischemia. After the clamp was released and the abdomen was closed, animals were subcutaneously injected with 100 ml/kg warm saline after the operation to assist in the maintenance of hydration. Animals were kept in an incubator (37°C) from the time of anesthetic administration until completely awake. Survival rates, serum creatinine (Scr) levels, and renal histopathology were assessed at the designated time points. The rat kidney fibrosis model was produced by 28 days after IRI.

### Generation of the Hypoxia/Reperfusion Cell Model

The renal tubular epithelial cell line, NRK-52E, was purchased from Procell (Wuhan, China) and cultured in Dulbecco’s modified Eagle’s medium (DMEM, Gibco, Waltham, MA, USA) supplemented with 10% fetal bovine serum (FBS), penicillin (100 U/ml), and streptomycin (100 mg/ml) and maintained at 37°C in a humidified atmosphere of 5% CO_2_. After reaching confluency >80%, the hypoxia/reperfusion (H/R) cell model was generated by replacing the culture medium with low glucose serum-free DMEM. Cells were incubated at 1% O_2_ and 5% CO_2_ balanced with N_2_ in a multigas incubator (APM-30D, ASTEC, Fukuoka, Japan) for 24 h. The normal medium was replaced and cultured for another 6 h at 37°C, 5% CO_2_ after induction of hypoxia. E_2_ (100 nM), ERα siRNA (100 nM), and plasmid encoding TGF-βRI (pCDNA-TGF-βR1) or empty vector (pCDNA) (1 μg) were transfected into the cells for 24 h before the induction of hypoxia. The cells were incubated for another 48 h. After treatment, cells were collected and harvested by homogenization for further analysis by Western blotting and quantitative PCR.

### Immunostaining of Fluorescence and Histochemistry

For fluorescence staining, the cell slices were permeabilized and incubated in a blocking solution. Next, the sections were incubated with primary antibodies at 4°C overnight. The slices were then incubated with secondary antibodies conjugated with Coralite 488/546 (Proteintech, China). Finally, DAPI was used for nuclear staining. The images were observed under a laser confocal microscope (710, Zeiss, Jena, Germany). For immunohistochemistry, paraffin-embedded kidney tissue samples were incubated at 72°C for 30 min. After the xylene was dewaxed, samples were rehydrated using gradient ethanol. Heat-induced sodium citrate antigen retrieval was performed at 95°C for 30 min using sodium citrate. The slices were incubated with secondary antibodies conjugated with HRP (Proteintech, China). After washing with PBS, DAB was used as the developing agent. Finally, samples were incubated with hematoxylin; thus, nuclear staining was performed. Images were captured by Nikon and analyzed using the Image J software.

### Renal Histopathology

Kidney tissues were fixed in 4% PFA for 24 h and embedded in paraffin. Masson and Sirius red staining procedures were performed to estimate the extent of renal fibrosis. Two renal pathologists, blinded to the experiment, quantified the staining in eight randomly selected fields. Data were analyzed using Image-Pro Plus software (Media Cybernetics, Rockville, MD, USA).

### TUNEL Staining

Apoptotic cell death was quantified using the TUNEL-FITC assay. Kidney sections were deparaffinized, hydrated, and treated with proteinase K solution in Tris-EDTA buffer. After pre-equilibration, strands of DNA were end-labeled by incubation with recombinant terminal deoxynucleotidyltransferase (rTdT) enzyme. The reaction was terminated upon the addition of saline sodium citrate buffer. After washing the nuclei, DAPI labeling was performed. The slides were mounted and observed using a fluorescence microscope (Carl Zeiss). In each section, apoptotic cells were counted in five randomly chosen nonoverlapping fields in the outer stripe of the renal medulla for each rat. Results were expressed as apoptotic cells per field.

### H&E Staining and Tubular Injury Score

Sections were washed in distilled water and nuclei were stained with alum hematoxylin. Samples were rinsed under running tap water, differentiated with 0.3% acid alcohol, and again rinsed under running tap water. Next, the sections were stained with eosin, dehydrated, made clear, and mounted. The tubular injury was scored based on the H&E staining. The scoring was performed by grading the tubular injury, epithelial cell apoptosis, intraluminal cast, and brush border loss in 10 randomly chosen nonoverlapping fields (×100 magnification). The lesions were graded on a scale of 0 to 4, wherein, 0 represented normal state; 1, indicating that the injury was less than 25% of the field for tubular injury; 2, indicating that the injury was between 25% and 50%; 3, the injury was between 50% and 75%, and 4 indicated extensive injury of more than 75%. Kidney fibrosis area fraction was quantified using Image J software was used to analyze 10 randomly chosen, nonoverlapping fields in each sample.

### RNA Isolation and RT-PCR

Total RNA was extracted using Trizol reagent (Gibco, Life Technologies, Darmstadt, Germany) according to the manufacturer’s protocol. A reverse transcriptase-polymerase chain reaction was performed after the RNA was amplified by reverse transcription (RT) using an Oligo (dT) primer (Yeasen, Shanghai, China). Specific cDNA products corresponding to the mRNA were amplified and detected by SYBR^®^Green Real-Time PCR (Yeasen, China). Amplification was performed on the ABI Stepone Plus platform. Primers sequences as follows: TGF-βRIforward: 5′-GTCAGCTGGCCTCGGTC-3′; reverse: 5′-ATGACAGTGCGGTTATGGCA-3′. TGF-β1 forward: 5′-ATACGCCTGAGTGGCTGTCT-3′; reverse: 5′-TGGGACTGATCCCATTGATT-3′. TNF-α forward: 5′-GAGGTCAACCTGCCCAAGTA-3′; reverse: 5′-GGGGGCTCTGAGGATTAGAC-3′. IL-1β forward: 5′-CTGTGACTCGTGGGATGATG-3′; reverse: 5′-GGGATTTTGTCGTTGCTTGT-3′. IL-6 forward: 5′-CCGGAGAGGAGACTTCACAG-3′; reverse: 5′-ACAGTGCATCATCGCTGTTC-3′.MCP-1 forward: 5′-TGATCCCAATGAGTCGGCTG-3′; reverse: 5′-TGGACCCATTCCTTATTGGGG-3′.

### Western Blot Analysis

Kidney cortical tissue samples (50 mg) were homogenized in lysis buffer and proteins were extracted and their levels were quantified using the Bicinchoninic Acid Protein Assay Kit (Beyotime, Shanghai, China). Samples were separated on the sodium dodecyl-sulfate polyacrylamide gels (10%) and transferred onto PVDF membranes. The membranes were incubated with primary antibodies. Protein bands were visualized by electrochemiluminescence, and protein detection was on the ECL Gel imager analysis system (JS-680D, Peiqing, Shanghai, China). The relative band intensity was quantified using an ImageJ system (NIH, Bethesda, MD, USA).

### Dual-Luciferase Reporter Assay

The procedure was performed according to the manufacturer’s instructions (RG088S, Beyotime, China). Briefly, HEK293T cells were transfected with ERα plasmid or its empty vector control in combination with pTGF-βR1-Luc reporter construct, pRL-TK, and E_2_ and incubated for 48 h. Next, the cells were lysed using the detection buffer for 15 min. The supernatant of the lysate was used to measure the expression of the luciferase reporter gene. The activity of pTGF-βR1-Luc was normalized with the transfection efficiency using the activity of Renilla luciferase (pRL-TK). To generate luciferase reporter plasmids of TGF-βRIpromoter, DNA fragments (predicted ERE sequence: TCAAATTTAGTCTCTGTAGCCTCGGTGC; Mut ERE sequence: TCTTAACTCATGACAGTACGTATCTCGGTGC) were synthesized and inserted into the pGL3 basic luciferase expression vector (Promega, Madison, WI, USA). HEK293T cells were transfected using luciferase reporter plasmids. The luciferase activity reflected the transcriptional activity of the target genes. To assay the luciferase activity, cell extracts were detected on the Dual-Luciferase Reporter (DLR) assay system (Promega).

### Chromatin Immunoprecipitation Assay

For the chromatin immunoprecipitation (ChIP) assay, the cells were treated as per the manufacturer’s instructions (P2078, Beyotime, China). The cross-linking between the DNA and proteins was accomplished using formaldehyde, and the reaction was terminated using glycine solution. Samples were washed thrice and incubated in cold PBS with PMSF. After centrifugation, the cell pellet was resuspended in SDS lysis buffer with PMSF and incubated on ice. After incubation, cell extracts were sonicated for 5–10 s and centrifuged. Liquid supernatant was transferred to a new tube and ChIP dilution buffer was added to it. An aliquot was reserved as the input, and the remainder was divided into immune-precipitate and incubated with control rabbit IgG or ERα (Cell Signaling) antibody and protein A+G beads, overnight at 4°C. After centrifugation, the pellet was rinsed once with the following solutions successively: low salt immune complex wash buffer, high salt immune complex wash buffer, and LiCl immune complex wash buffer. Finally, the pellet was rinsed twice with TE buffer. A total of 250 µl of the elution buffer was added to the pellet and vortexed to mix. For reverse cross-linking, samples were incubated at 65°C for 4 h. Next, 10 µl EDTA, 20 µl Tris, and 1 µl proteinase K were added, vortexed, and incubated at 45°C for 60 min. After isolation with phenol/chloroform buffer, 20 µg glycogen was added to the pellet, along with NaAc and absolute ethanol. After vortexing, the mixtures were incubated at −80°C for at least 8 h and centrifuged at 14,000×*g* for 10 min at 4°C. The DNA in the pellet was resuspended in TE buffer. Primers used for ChIP PCR were as following: ERE1 forward: 5′-TCTTGGCATGTGGCTGTAAC-3′; reverse: 5′-GGCTACCCGACAAGAATGC-3′. ERE2 forward: 5′-CTTGCCGAGGAAGTACAAGG-3′; reverse: 5′-CGCTTGAGCAAGTCCTAACC-3′. ERE3 forward: 5′-GAATCCCCCATCATTCAAAA-3′; reverse: 5′-CCTCAAATTCCAGTCCCAAA-3′. ERE4 forward: 5′-GCGTGGTTAGAGGCAGAAGT-3′; reverse: 5′-CTCCGGCCCTTTGTAACTG-3′. Products were analyzed by gel after PCR.

### Flow Cytometry to Analyze Cell Apoptosis Rates

Flow cytometric analysis was performed to evaluate the apoptotic cell rates after H/R. Before exposure to H/R, NRK-52E cells were first transfected with TGF-βRI plasmid or control vector, or ERα siRNA or NC siRNA, and treated with or without E_2_ (100 nM) for 24 h. After treatment, the NRK-52E cells were trypsinized and centrifuged at 1,500 rpm for 5 min. According to the manufacturer’s instructions, 400 µl binding fluid contained 10 µl Annexin V was added to 10^6^ cells/ml. Cells were restained with 10 µl PI for 10 min and gently placed at 4°C in the dark before immediately being analyzed using a laser eight-color flow cytometer (FACSVerse, BD, Piscataway, NJ, USA) and quantified using FlowJo 7.6 software 21.

### Statistical Analysis

All results were expressed as means ± SEM. An unpaired *t*-test was used to assess statistical significance between the two groups. In multiple comparisons of three or more groups, the statistical significance was assessed by one-way or two-way ANOVA followed by a *post-hoc* test (Bonferroni’s method). Statistics were computed using Graphpad Prism 6 (GraphPad Software). A *p* < 0.05 was considered statistically significant.

## Results

### Renal Injury in Female Rats Suffered Was Less Extensive Than That in Male Rats After IRI

**Figure 1:**
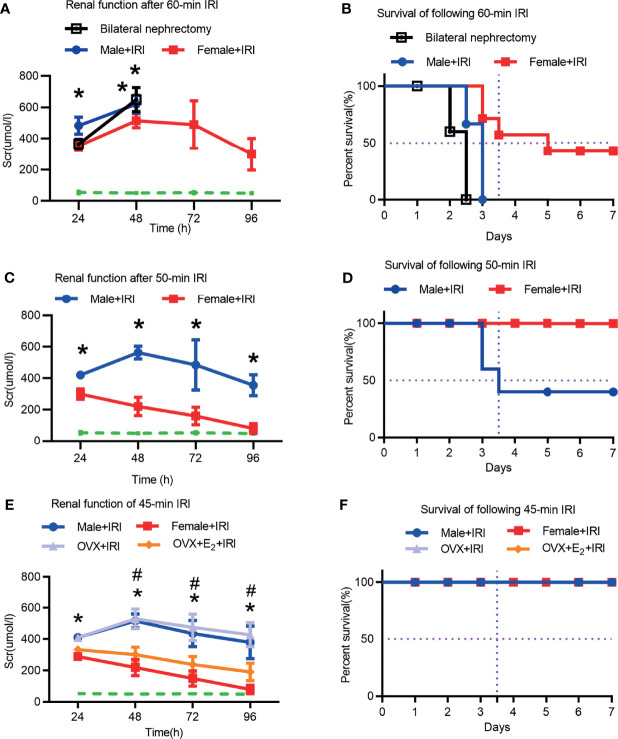
Renal function and survival rates were assessed in male and female rats. **(A)** Scr levels were measured in male and female rats for 4 consecutive days after 60 min of ischemia or in male rats undergoing bilateral nephrectomy (bilat. neph). Two-way ANOVA followed by Bonferroni’s *post-hoc* test, ^*^ *p* < 0.05, vs. female+IRI. The dashed line shows the baseline Scr level, *n* = 6. **(B)** Survival rates of male and female rats subjected to 7 consecutive days of ischemia for 60 min. **(C)** Scr levels in male and female rats subjected to 4 consecutive days of ischemia for 50 min. Two-way ANOVA followed by Bonferroni’s *post-hoc* test, ^*^ *p* < 0.05, vs. female+IRI. **(D)** Survival rates of male and female rats after seven consecutive days of ischemia for 50 min. **(E)** Scr levels in male and female rats, OVX rats, and OVX rats that received E_2_ after being subjected to 45 min of ischemia for four consecutive days. Two-way ANOVA followed by Bonferroni’s *post-hoc* test, ^*^ *p* < 0.05, vs. female+IRI; ^#^ *p* < 0.05, OVX+IRI vs. OVX+E_2_+IRI. **(F)** Survival rates of male and female rats, OVX rats, and OVX+E_2_ rats that were subjected to 45 min of IRI for seven consecutive days, *n* = 8 for each group. The data are presented as the mean ± SEM.

First, ischemia times from 45 to 60 min were evaluated to determine the optimal time. The serum creatinine (Scr) levels of bilateral nephrectomy rats were used to determine lethality in the model. As shown in **Figures 1A, B**, the Scr levels in male rats after 60 min of ischemia were as high as those in rats with bilateral nephrectomy, suggesting that 60 min of ischemia was lethal and not an ideal model for this study. Next, after 50 min of ischemia, the Scr concentrations in the female+IRI were markedly lower than those in the male+IRI, indicating less injury in female rats. However, the male rat survival rate after 50 min of ischemia was only 50%, which was not ideal for this study (**Figures 1C, D**). Finally, 45 min of ischemia was shown to be appropriate for the experimental animal model because the Scr values differed significantly between males and females and because the survival rates were improved compared with those at the 50-min time point (**Figures 1E, F**). Therefore, we utilized 45 min of ischemia to induce the rat IRI model in this study. Next, we found that OVX+E_2_+IRI effectively attenuated the Scr concentrations compared with those in the OVX+IRI (**Figure 1E**), providing evidence of the protective effect of the ovary during IRI.

### Estrogen-Mediated Protection of Female Rats During IRI

**Figure 2:**
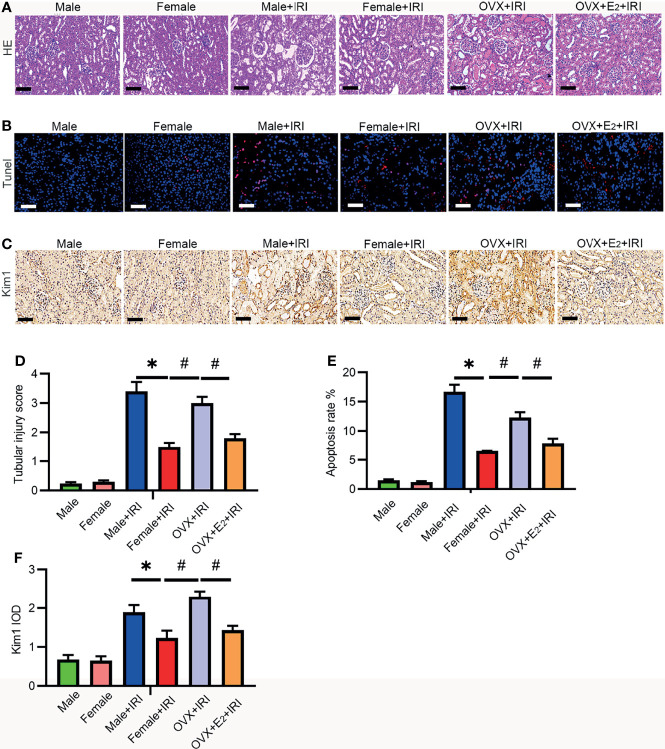
E_2_ ameliorated IRI-induced kidney injury. **(A)** After 45 min of ischemia followed by 24 h of reperfusion, the tubular injury levels in female rat kidneys were examined by HE staining **(A)** and statistically analyzed **(D)**. One-way ANOVA followed by Bonferroni’s *post-hoc* test, ^*^ *p* < 0.05, vs. female+IRI. ^#^ *p* < 0.05, vs. OVX+IRI. Bar = 100 μm. **(B)** Apoptotic cells in kidney tissues were examined by TUNEL-FITC staining **(B)** after 45 min of ischemia followed by 24 h of reperfusion and statistical analysis **(E)**. One-way ANOVA followed by Bonferroni’s *post-hoc* test, ^*^ *p* < 0.05, vs. female+IRI. ^#^ *p* < 0.05, vs. OVX+IRI. Bar = 100 μm. **(C)** Kidney injury was examined by IHC with a Kim-1 antibody after 45 min of ischemia followed by 24 h of reperfusion and statistical analysis **(F)**. Bar = 100 μm. One-way ANOVA followed by Bonferroni’s *post-hoc* test, ^*^ *p* < 0.05, vs. female+IRI. ^#^ *p* < 0.05, vs. OVX+IRI, *n* = 6 in each group. The data are presented as the mean ± SEM.

To investigate whether and how estrogen is involved in protection against IRI, female rats were subjected to ischemia after OVX. Furthermore, to determine the role of estrogen in IRI, OVX rats were administered E_2_ before IRI. As shown in **Figure 2**, the kidney tubular injury scores (HE staining) (**Figures 2A, D**) of male and OVX rats subjected to IRI were increased compared with those of normal female rats. Next, Kim-1 staining (**Figures 2C, F**) revealed that the kidney tissues of male and OVX rats were damaged to a greater extent than those of normal females, and TUNEL staining revealed more apoptotic cells in the male and OVX groups than in the normal females (**Figures 2B, E**). In contrast, supplementation with E_2_ reversed the decreased tubular injury scores and the apoptotic cell and kidney damage, suggesting that E_2_ attenuated injury induced by IRI.

### E_2_ Protected Against IRI-Induced Renal Fibrosis in Female Rats

**Figure 3:**
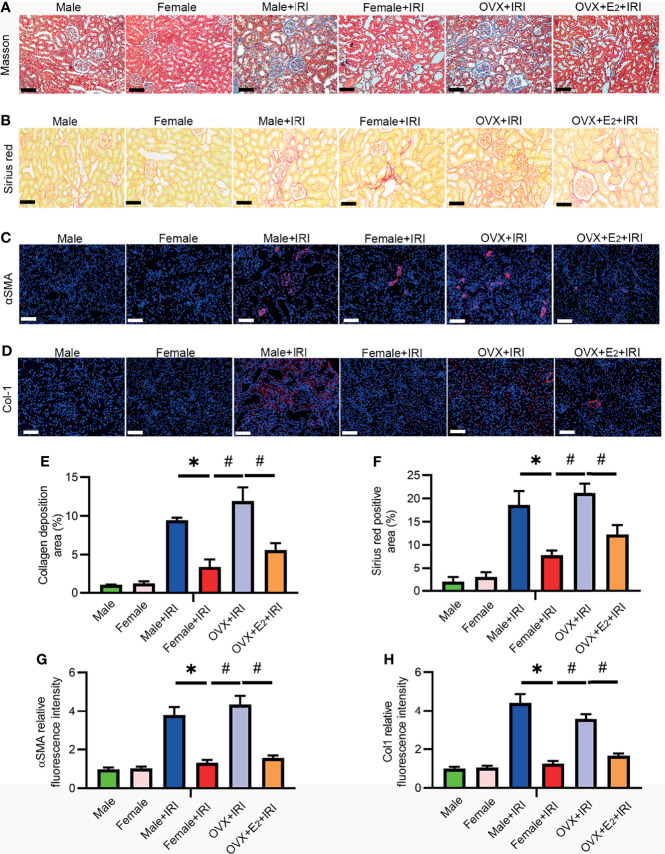
E_2_ attenuated renal fibrosis induced by IRI. **(A)** Representative sections of kidney tissues from female rats stained with Masson’s trichome **(A)** to examine changes in interstitial fibrosis (collagen stained in blue) and a histogram showing the statistical analysis **(E)**. One-way ANOVA followed by Bonferroni’s *post-hoc* test, ^*^ *p* < 0.05, vs. female+IRI. ^#^ *p* < 0.05, vs. OVX+IRI. Bar = 100 μm. **(B)** Representative sections of kidney tissues from different rats stained with Sirius red **(B)** and a histogram showing the statistical analysis **(F)**. One-way ANOVA followed by Bonferroni’s *post-hoc* test, ^*^ *p* < 0.05, vs. female+IRI. ^#^ *p* < 0.05, vs. OVX+IRI. Bar = 50 μm. **(C)** Immunofluorescence staining of α-SMA (red) and DAPI (blue) **(C)** in representative sections of kidney tissues from female rats and a histogram showing the statistical analysis **(G)**. One-way ANOVA followed by Bonferroni’s *post-hoc* test, ^*^ *p* < 0.05, vs. female+IRI. ^#^ *p* < 0.05, vs. OVX+IRI. Bar = 100 μm. **(D)** Immunofluorescence staining of Col1 (red) and DAPI (blue) **(D)** in representative sections of kidney tissues from female rats and a histogram showing the statistical analysis **(H)**. One-way ANOVA followed by Bonferroni’s *post-hoc* test, ^*^ *p* < 0.05, vs. female+IRI. ^#^ *p* < 0.05, vs. OVX+IRI. Bar = 100 μm, *n* = 6 in each group. The data are presented as the mean ± SEM.

Renal fibrosis is the most critical pathophysiological process responsible for the loss of kidney function and the core cause of the high morbidity and mortality rates associated with AKI and CKD (30). Masson’s trichrome and Sirius red staining are widely used in combination to measure tissue fibrosis, and fibrosis severity is assessed based on the average of semiquantitative scores of collagen staining (blue and red). Masson’s trichome (**Figures 3A, E**) and Sirius red staining (**Figures 3B, F**) revealed more collagen deposition in the OVX+IRI than in the female+IRI, and supplementation with E_2_ effectively reduced collagen accumulation. Moreover, several fibrotic markers, including α-SMA and collagen 1 (Col1), were also examined by immunohistochemistry to analyze the severity of renal fibrosis. Consistent with the previous results, kidneys of the female+IRI showed less positive staining for α-SMA (**Figures 3C, G**) and ColI (**Figures 3D, H**) than the kidneys of OVX+IRI rats, and this positive staining decreased after E_2_ supplementation in OVX rats. These results suggested that the administration of E_2_ ameliorated fibrosis after IRI in females.

### E_2_ Exerted a Protective Effect by Inactivating TGF-βRI

**Figure 4:**
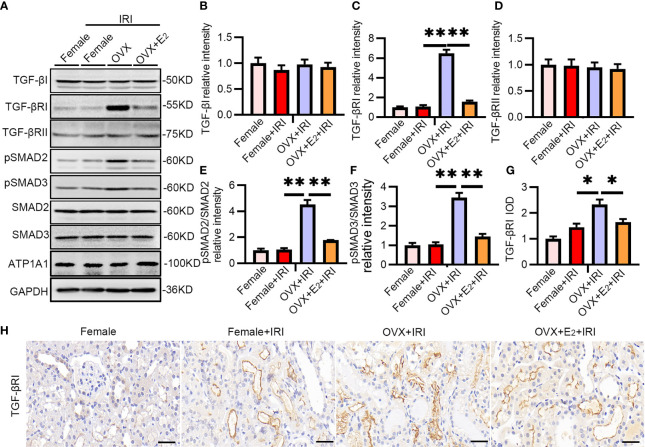
The TGF-βRI-SMAD pathway was activated in OVX rats after renal IRI. **(A)** After 45 min of ischemia followed by 24 h of reperfusion, female and OVX rat kidneys were homogenized and analyzed by Western blotting using TGF-βRI-PSMAD2/3 antibodies **(A)** and subjected to statistical analysis **(B–F)**. **(H)** TGF-βRI expression in kidney tissue as determined by IHC **(H)** and statistical analysis **(G)**. Bar = 100 μm. One-way ANOVA followed by Bonferroni’s *post-hoc* test, ^*^ *p* < 0.05, vs. OVX+IRI; ^**^ *p* < 0.01, vs. OVX+IRI, *n* = 6 in each group. The data are presented as the mean ± SEM.

To further examine the effect of estrogen, OVX rats and OVX rats pretreated with E_2_ were subjected to IRI, and the TGF-βRI-SMAD pathway was examined. As expected, the levels of TGF-βRI, pSMAD2, and pSMAD3 were substantially upregulated after IRI in OVX rats compared with female rats (**Figures 4A–F**). Immunohistochemistry staining confirmed the increased positive staining of TGF-βRI in the tubular cells of kidneys from OVX rats (**Figures 4G, H**). These results provided evidence that females suffered less IRI injury because estrogen exerted a protective effect by inactivating the TGF-βRI-SMAD pathway.

### E_2_ Reduced the Expression of TGF-βRI *via* the Nuclear Transcription Factor ERα

**Figure 5:**
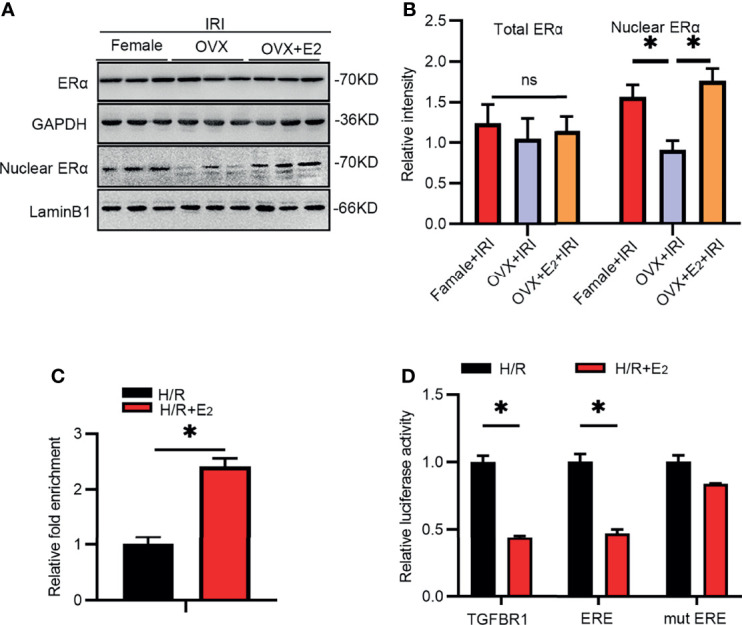
IRI activated TGF-βRI by suppressing ERα. **(A)** Total and nuclear extracts from kidney tissues of rats in the different groups as analyzed by antibodies against ERα **(A)** and a histogram showing the statistical analysis **(B)**. One-way ANOVA followed by Bonferroni’s *post-hoc* test, ^*^ *p* < 0.05, vs. OVX+IRI. **(C)** ChIP was used to analyze the predicted binding sites in the promoter region of TGF-βRI after treatment with H/R with or without E_2_ in NRK-52E cells for 24 h. Unpaired *t*-test, ^*^ *p* < 0.05, vs. H/R+E_2_. **(D)** Luciferase activity after transfection of pGL3-basic plasmids encoding the TGF-βRI promoter, ERE, or mut ERE sequence after treatment with H/R with or without E_2_ for 24 h. Unpaired *t*-test, ^*^ *p* < 0.05, vs. H/R+E_2_, *n* = 3 in each group. ns, non significance. The data are presented as the mean ± SEM.

Accumulating evidence suggests that ERα is a transcriptional regulator that activates or inhibits the expression of some genes at the mRNA level. On the other hand, E_2_ deficiency often results in a decrease in Erα expression. The total ERα protein level did not change after IRI in female rats (**Figures 5A, B**). However, analyses of nuclear kidney tissue extracts showed that the level of Erα, but not ERβ (data not shown), was obviously reduced in the kidneys of OVX rats compared with female rats, as determined by Western blot analysis. This decrease was ameliorated by E_2_ supplementation in OVX rats.

As predicted by JASPAR, several ERE binding sites resided in the promoter region of TGF-βRI, suggesting potential regulation between ERα and TGF-βRI. Thus, we constructed plasmids containing these ERE sequences and examined their binding with ERα using a dual luciferase reporter gene assay. As revealed by ChIP (**Figure 5C**), the binding of ERα to the ERE sequence was increased after E_2_ supplementation and hypoxia/reoxygenation (H/R) in NRK-52E cells. In addition, the luciferase activity (**Figure 5D**) at the predicted binding site was substantially inhibited after E_2_ treatment compared with that in the H/R group, suggesting that Erα binds to the ERE of TGF-βRI. The above results suggested that the *TGF-βRI* promoter ERE is a region in which ERα interacts with and inhibits its expression.

### TGF-βRI Knockdown Protected NRK-52E Cells From Hypoxic Injury

**Figure 6:**
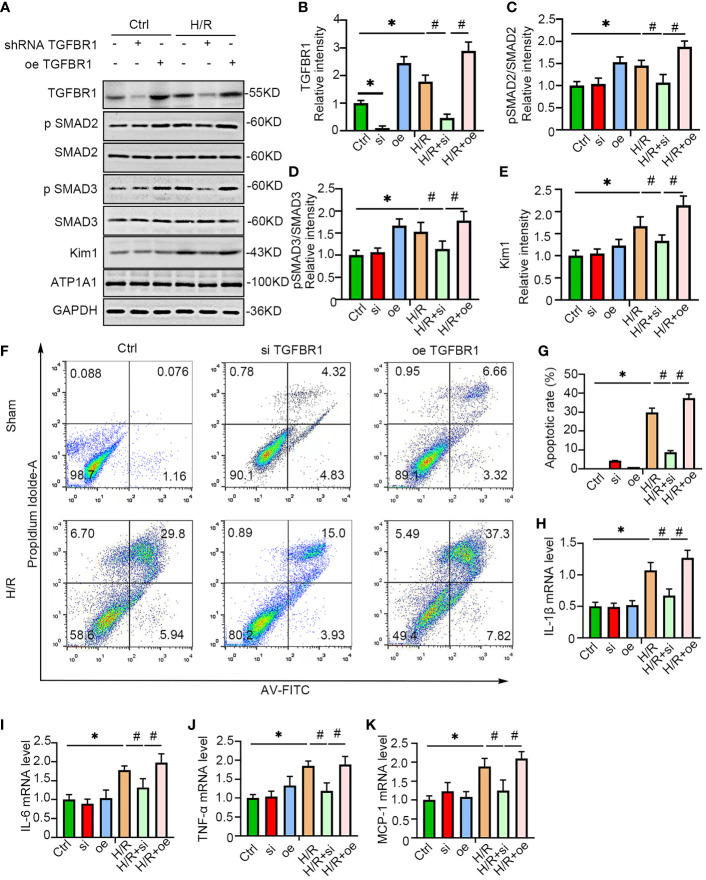
TGFBR1 knockdown protected NRK-52E cells from H/R. **(A)** TGFBR1 was overexpressed or knocked down in NRK-52E cells, which were then stimulated with H/R. The cells were harvested and examined with antibodies related to the TGFBR1-SMAD pathway and Kim-1 **(A)**, and the statistical analysis results are presented as a histogram **(B–E)**. One-way ANOVA followed by Bonferroni’s *post-hoc* test, ^*^ *p* < 0.05, vs. Ctrl. ^#^ *p* < 0.05, vs. H/R+si. **(F)** Apoptotic NRK-52E cells were examined using flow cytometry after H/R and overexpression or knockdown of TGFBR1 **(F)**, and the statistical analysis results are presented as a histogram **(G)**. One-way ANOVA followed by Bonferroni’s *post-hoc* test, ^*^ *p* < 0.05, vs. Ctrl. ^#^ *p* < 0.05, vs. H/R+si. **(H–K)** qPCR was used to measure the mRNA levels of IL1-β **(H)**, IL-6 **(I)**, TNF-α **(J)**, and MCP-1 **(K)** in NRK-52E cells after H/R and overexpression or knockdown of TGFBR1. One-way ANOVA followed by Bonferroni’s *post-hoc* test, ^*^ *p* < 0.05, vs. Ctrl; ^#^ *p* < 0.05, vs. H/R+si, *n* = 3 in each group.The data are presented as the mean ± SEM.

To further verify the role of TGF-βRI in the induction of IRI, gain and loss of function were examined by the overexpression and shRNA-mediated knockdown of TGF-βRI, respectively, in NRK-52E cells under hypoxic conditions. Consistent with the animal study, TGF-βRI overexpression substantially augmented the downstream expression of pSMAD2/3 under hypoxia. In contrast, knockdown of TGF-βRI expression ameliorated the activation of pSMAD2/3 and Kim-1 upon the induction of hypoxia (**Figures 6A–E**). Flow cytometry also indicated that the number of apoptotic cells was increased under H/R conditions, and this increase was ameliorated by TGF-βRI knockdown and enhanced by TGF-βRI overexpression (**Figures 6F, G**). Moreover, the levels of the proinflammatory factors IL-1β, IL-6, TNF-α, and MCP-1, which were upregulated by hypoxia, were decreased by TGF-βRI knockdown (**Figures 6H–K**), suggesting that TGF-βRI plays a role in regulating inflammation in hypoxia.

### Erα Knockdown Eliminated the Protection of NRK-52E Cells From Hypoxia

**Figure 7:**
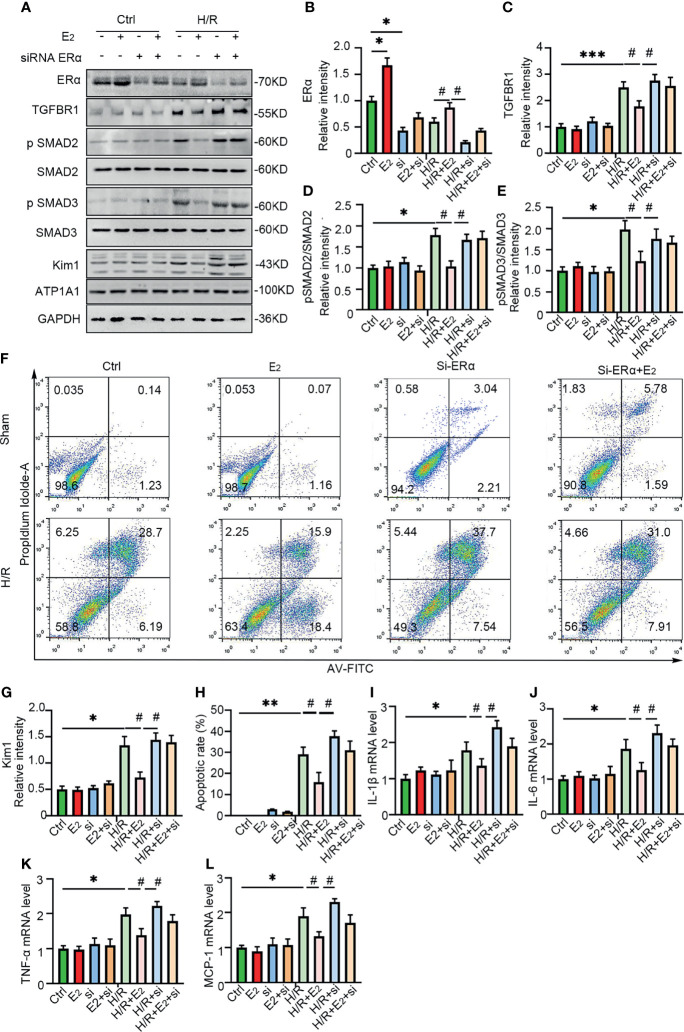
ERα exerted protective effects on H/R-induced NRK-52E cells. **(A)** ERα was first activated or knocked down in NRK-52E cells, which were then stimulated with H/R. The cells were harvested and examined with ERα, Kim-1, and TGFBR1-SMAD pathway antibodies **(A)**, and the statistical analysis results are presented as a histogram **(B–E, G)**. One-way ANOVA followed by Bonferroni’s *post-hoc* test, ^*^ *p* < 0.05, vs. Ctrl. ^#^ *p* < 0.05, vs. H/R+E_2_. **(F)** Apoptotic NRK-52E cells were examined by flow cytometry after H/R and activation or knockdown of ERα **(F)**, and the statistical analysis results are presented as a histogram **(H)**. **(I–L)** qPCR was performed to examine the mRNA levels of IL-1β **(I)**, IL-6 **(J)**, TNF-α **(K)**, and MCP-1 **(L)** in NRK-52E cells after H/R and stimulation or knockdown of ERα. One-way ANOVA followed by Bonferroni’s *post-hoc* test, ^*^ *p* < 0.05, vs. Ctrl; **p < 0.01, vs. Ctrl; ***p < 0.001, vs. Ctrl. ^#^ *p* < 0.05, vs. H/R+E_2_, *n* = 3 in each group. The data are presented as the mean ± SEM.

**Figure 8:**
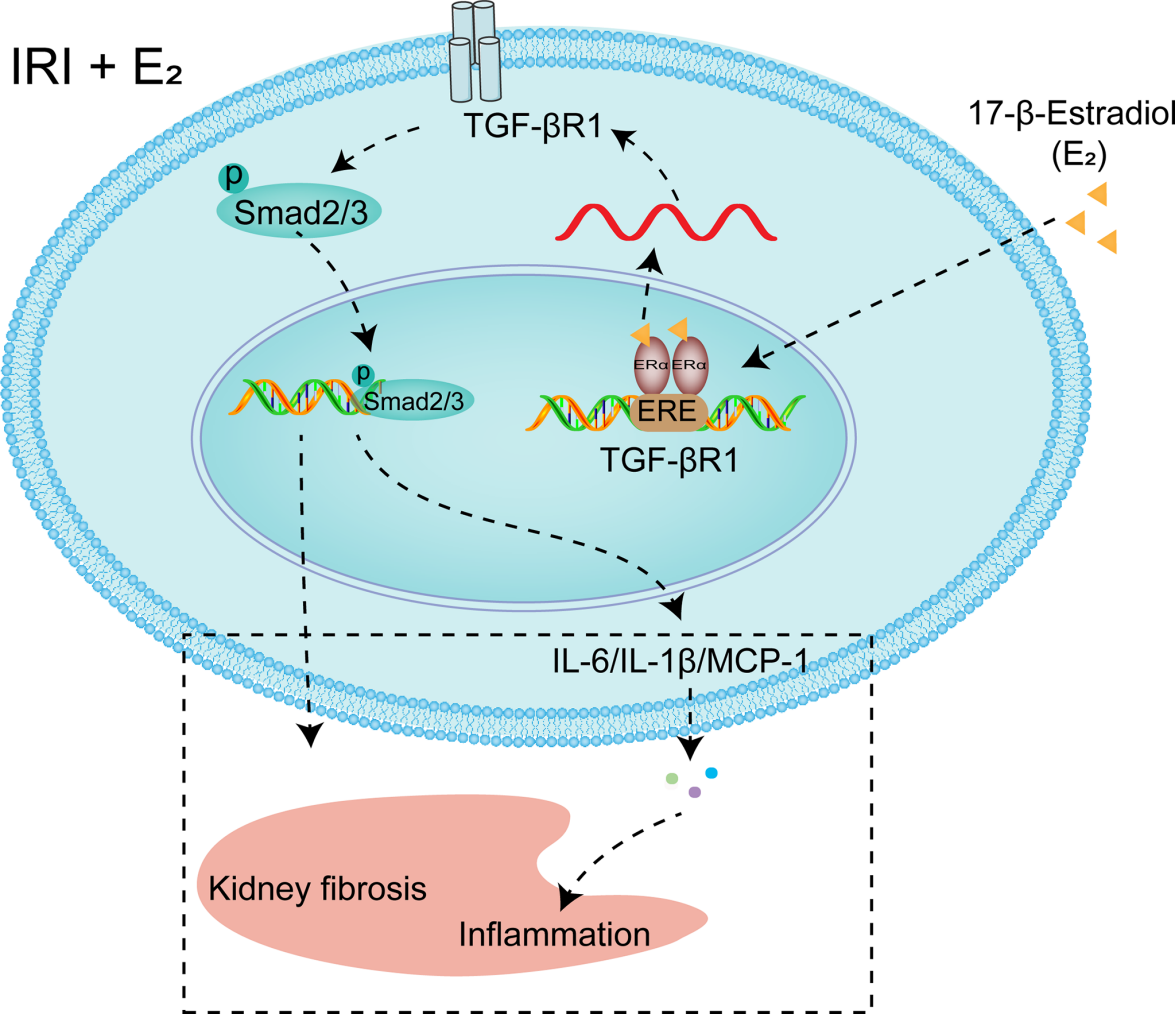
Diagram showing the mechanism by which E_2_ negatively regulates TGF-βRI gene transcription. Under the condition of IRI, a decrease in the E_2_ level by OVX reduces the transcriptional inhibition of TGF-βRI, and activating TGF-βRI activates pSMAD2/3, thus promoting the release of proinflammatory factors. After supplementation, E_2_ enters the nucleus, binds to ERα, binds to the ERE of the TGF-βRI promoter, and represses its transcription.

To further confirm the role of ERα in the regulation of TGF-βRI under IRI conditions, gain and loss of function were examined by E_2_ stimulation and siRNA-mediated knockdown of ERα in NRK-52E cells under hypoxic conditions. As shown in **Figure 7**, ERα knockdown robustly blocked the activation of pSMAD2/3 after hypoxia, while ERα stimulation ameliorated the activation of pSMAD2/3 and Kim-1 induced by hypoxia (**Figures 7A–E, G**). Flow cytometry revealed that E_2_ supplementation ameliorated apoptosis under H/R conditions, but this restoration was inhibited by ERα knockdown (**Figures 7F, H**). In addition, the levels of the proinflammatory factors IL-1β, IL-6, TNF-α, and MCP-1 (**Figure 7I–L**), which were upregulated by hypoxia, were decreased by supplementation with E_2_, suggesting the protective effect of E_2_ under hypoxic conditions. In conclusion, ERα, as a transcriptional repressor, binds to the ERE sequence of TGF-βRI and inhibits its expression, downregulates pSMAD2/3 activation, reduces the production and release of proinflammatory factors, and contributes to the protection of renal IRI (**Figure 8**).

## Discussion

E_2_ is an established renoprotective agent in various animal models of kidney injury (31). Females are more tolerant to AKI than males, and this protection diminishes with age because postmenopausal women are at an increased risk for AKI (32). In this study, 45 min of renal ischemia followed by 24 h of reperfusion resulted in AKI, as reflected by marked increases in Scr levels and pathological changes in male and OVX rat kidneys as well as by increased TUNEL-positive staining, and these effects were reversed by E_2_ supplementation. In addition, a significant decrease in the nuclear ERα level and increases in the levels of TGF-βRI and downstream pSMAD2/3 were observed in OVX rats, and these alterations were reversed after E_2_ supplementation. Furthermore, ERα bound to the ERE promoter sequence of TGF-βRI and negatively regulated its mRNA expression. Moreover, knockdown of TGF-βRI and ERα in NRK-52E cells blocked and exacerbated H/R-induced injury, respectively, further demonstrating the role of ERα-TGF-βRI in IRI-induced injury.

Previous mouse studies indicated that longer renal pedicle clamp times (30 min or longer), which are designed to induce severe AKI, induce more extensive renal tubular injury than shorter clamp times, which are designed to induce mild-to-moderate AKI (33). In addition, many critical issues other than the ischemia time following IRI can influence the severity of AKI, such as strain, sex, age, and weight (6, 34, 35). Although longer ischemia times induce more significant pathological changes, they are also associated with higher mortality (36). To establish an ideal AKI rat model, ischemic times ranging from 45 to 60 min after occlusion were examined, and the survival rates and renal injuries were assessed. We observed low survival rates as well as high Scr concentrations in male rats subjected to 50 and 60 min of ischemia, implying that the kidney damage caused by these two ischemic times was too severe. The survival rates of male and female rats subjected to 45 min of ischemia did not differ, and the Scr concentration was substantially enhanced compared with that in the normal group, suggesting that the 45-min ischemia time was not lethal and caused significant renal injury, thus making it an ideal model of IRI. Therefore, we pretreated OVX rats with E_2_ to investigate its protective effect against IRI. As expected, the administration of E_2_ to OVX rats effectively decreased the Scr concentration compared with that in rats not treated with E_2_. Finally, 45 min of warm ischemia was demonstrated to be the most ideal length of time due to the observance of classical IR features and to the lack of lethal effects. Consistent with previous studies (37), 45 min of warm ischemia resulted in significant, recoverable injury and was recommended for the investigation of renal reperfusion injury. The level of another kidney injury marker, Kim-1, was substantially enhanced after 45 min of IR, as revealed by immunohistochemistry staining. However, kidney staining for Kim-1 was slightly but not significantly darker in the OVX group than in the male group, implying that female rats suffered more kidney damage after the protective effect of estrogen was lost. These results may be ascribed to the fact that OVX has been demonstrated to accelerate kidney disease only in various animal models without IRI (38, 39).

Renal fibrosis is a final common stage of CKD (40) and is mainly mediated by the TGF-β1-SMAD pathway. Conditional knockout of the TGF-βRII/Smad2 pathway reportedly exerts a protective effect on acute renal injury by alleviating cell necroptosis, apoptosis, and inflammation (41). Overexpression of TGF-βRII increases the expression of collagen I and α-SMA, which are closely related to renal fibrosis (42). However, the role of TGF-β1 in renal IRI is still unclear. Some researchers have found that TGF-β1 plays a protective role in acute injury, while others have shown that the accumulation of TGF-β1 induces AKI (14, 15). These discrepancies may be correlated with cell type-specific TGF-β1 levels and different types of AKI models. In this study, significant upregulation of TGF-βRI and pSMAD2/3, but not TGF-β1 or TGF-βRII, was observed in OVX rats after the induction of IRI. Activation of TGF-βRI alone mimics pathological alterations in AKI and has been verified by a previous study, which generated transgenic mice expressing ligand-independent constitutively active TGF-βRI kinase specifically in tubular epithelial cells (TECs). This transgenic mouse was characterized by marked tubular cell apoptosis and necrosis, reduced renal function, oxidative stress, and interstitial accumulation of inflammatory cells (15), all of which are pathological features of AKI. Furthermore, overexpression of TGF-βRI in NRK-52E cells activated pSMAD2/3 and downstream inflammatory effectors, such as MCP-1, providing evidence that the activation of TGF-βRI was sufficient to promote inflammation and oxidative stress. Normally, activation of the TGF-β1-SMAD pathway, including the binding of TGF-β1 to TGF-βRII, promotes the dimerization of TGF-βRII with TGF-βRI, induces the transphosphorylation of TGF-βRI, and activates the phosphorylation of the Smad2/3/4 protein at the C-terminus (43). However, we observed only the augmentation of TGF-βRI at the protein and mRNA levels and failed to observed effects on TGF-β1 or TGF-βRII, indicating that IRI does not activate classic TGF-β1-SMAD pathways. In addition to renal fibrosis, other fibrotic diseases are also less common in females than males. In addition to renal fibrosis, other fibrotic diseases are also less common in females than males. For example, the lower incidence rate of liver fibrosis in women compared with men may be attributed to genes associated with liver fibrosis responding differently to the loss of Ezh1/Ezh2 in the male liver (44). Kalafatis et al. showed that the pulmonary function of female patients with idiopathic pulmonary fibrosis (IPF) was more preserved than that of men at the time of inclusion, while men had significant cardiovascular complications (45).

Estrogen is a primary sex hormone that is synthesized in the ovary and controls reproductive functions in females (46). In males, it is synthesized *via* the conversion of testosterone to E_2_ by the aromatase enzyme (47). E_2_ is the predominant and most active form of estrogen, and estrogen has been shown to be a renoprotective agent in various studies (48, 49). ERα and ERβ are present in various regions of the nephron, including podocytes, distal convoluted tubules, and the ascending loop of Henle, suggesting their involvement in the maintenance of renal homeostasis (50). Estrogen exerts protective effects on renal damage in various models because of its anti-inflammatory and antioxidant activity (51). In this study, E_2_ supplementation protected against renal IRI by decreasing the levels of Scr, apoptotic cells, renal fibrosis, and oxidative stress. Estrogen inhibits inflammation through its receptors ERα, ERβ, and GPER (52), and a clinical study comparing the expression of ERα in the renal tissues of patients with immunoglobulin A nephropathy (IgAN) suggested that the expression of ERα in the glomeruli of IgAN renal tissue decreased gradually as the severity of renal dysfunction increased, as indicated by the estimated glomerular filtration rate (eGFR), Scr clearance rate, and pathological grade (53). More importantly, several ER response elements are reported to exist in the promoter region of the TGF-βRI gene, and E_2_ treatment reduces the mRNA expression of TGF-βRI in MC3T3−E1 cells (29). As male and OVX rats suffered more damage after IRI than normal female rats, E_2_ is hypothesized to exert a protective effect during IRI. Another report indicated substantially lower cortical Erα levels in the kidneys of male and OVX female rats than in those of normal females (54); thus, we speculate that ERα mediates the effect of E_2_ on TGF-βRI. In this study, we observed decreased ERα expression after IR in animals and cell models, and pretreatment with E_2_ or ERα overexpression ameliorated these injuries, providing evidence that ERα protects the kidney from IRI. The protein level of ERα and the concentration of E_2_ were also substantially downregulated in female rats after OVX. Furthermore, we found that this protection was due to the ERα-mediated transcriptional regulation of TGF-βRI, which is involved in renal fibrosis, oxidative stress, and inflammation. This assumption was further confirmed by dual luciferase and ChIP assays, which indicated that ERE existed in the promoter region of *TGF-βRI* and was transcriptionally inhibited by ERα. An *in vitro* cell model further confirmed the involvement of ERα and TGF-βRI in kidney injury following IR. As experiments were not carried out in ERα-knockout mice, we could not completely rule out other E_2_-associated receptors playing roles in the process of IRI. In addition, the underlying mechanism by which IR reduces the level of ERα is still not clear, and the specific role of estrogen and its receptors in inhibiting inflammation in the context of cell fate and human diseases such as AKI, cancers, cardiovascular diseases, and nervous system diseases remains unclear (52). In addition to the TGF-βRI-SMAD pathway, other pathways may be involved in ERα regulation, such as JAK/STAT, a proinflammatory pathway that could also be inhibited by E_2_ (55).

Currently, the number of patients requiring RRT worldwide is estimated to be between approximately 4.902 million (95% CI, 4.438–5.431 million) and 9.701 million (95% CI, 8.541–1.102 million), illustrating the magnitude of the disease burden of end-stage renal disease (ESRD) (56). IRI is unavoidable in kidney transplantation and is considered to be one of the most important mechanisms underlying nonfunction or delayed function after transplantation (57). The findings of the present study performed using cell and animal studies are highly valuable for preventing IRI after kidney transplantation. Based on the findings of the current study, the expression of ERα or TGFBR1 in the kidney can feasibly be altered to reduce the activation of the SMAD pathway, thereby reducing the production of inflammatory mediators in the context of renal fibrosis. Patients requiring kidney transplantation, for example, can be administered E_2_ in advance to activate ERα or administered TGF-BR1 antagonists to inhibit the SMAD pathway and thereby reduce inflammatory mediator production and renal fibrosis.

## Conclusion

In conclusion, this study revealed that the ERα receptor in the rat kidney was downregulated upon IRI, which dysregulated TGF-βRI and ultimately resulted in the activation of SMAD2/3 and the production and release of downstream inflammatory factors, resulting in severe renal IRI. Administration of E_2_ ameliorated inflammation *via* activation of the ERα receptor, and this effect was abolished by ERα knockdown or TGF-βRI overexpression, suggesting that the ERα receptor TGF-βRI mediates the renoprotective function of E_2_. Our results shed light on the ERα-TGF-βRI pathway as a potential target for the prevention of renal IRI.

## Data Availability Statement

The original contributions presented in the study are included in the article/supplementary material. Further inquiries can be directed to the corresponding author. The datasets generated for this study can be found in here https://www.jianguoyun.com/p/DWAQMawQutmNChjItKEE, access password: zJFG5T.

## Ethics Statement

The animal study was reviewed and approved by the Experimental Animal Committee of Wuhan University (Approval No. 2018121).

## Author Contributions

LR, FL, and ZD performed experiment operations, data analysis, and manuscript writing; helped in cell culture; and performed part of Western blot. YW designed the experiment. YX, SZ, QM, XB, ZL, and QY gave advice and assisted in the experiments and conceived and designed the study. All authors reviewed and approved the manuscript.

## Funding

This study was supported by the grants from the Scientific research Program of Hubei Provincial Health Commission (No. WJ2021Z005).

## Conflict of Interest

The authors declare that the research was conducted in the absence of any commercial or financial relationships that could be construed as a potential conflict of interest.

The handling editor NG declared a past coauthorship with one of the authors YW.

## Publisher’s Note

All claims expressed in this article are solely those of the authors and do not necessarily represent those of their affiliated organizations, or those of the publisher, the editors and the reviewers. Any product that may be evaluated in this article, or claim that may be made by its manufacturer, is not guaranteed or endorsed by the publisher.
